# Squill

**Published:** 1845-04

**Authors:** 


*Squill.*—Squill obviates some of the ill effects of opium. If an aperient be added besides, the good effects of opium may often be obtained when it would otherwise be inadmissible.

℞ Pulv. opii; pulv. scillæ, aa gr. iii.; aloes; conservæ rosæ, aa gr. ix. Fiat mass. divid. in pill. vi. æquales. Sign, opiate pills, two at bed-time.

We have remarked very beneficial results in cases of chronic ill health with tendency to dryness of the skin, from the continued use of such combinations as the following. Besides its diaphoretic effect, it should be regarded as alterative.

℞ Resinæ guaiaci; extract. gentianæ, aa ʒi.; pulv. ipecacuanhæ, gr. xv. Ft. mass. divid. in pill. xxx. æquales. Sign, tonic alterative pills, two twice a day.

Or, if an aperient be required, aloes may be substituted for the gentian, and then the combination will somewhat resemble the pulv. aloes comp. of the London Pharmacopœia.

A simple and very effectual treatment of the less severe bowel complaints, so apt to prevail at this season, is the following:

℞ Sulph. magnesiæ, ℥ss; aquæ menthæ, ℥vi. Solve. Sign, a wine-glassful to be used at intervals of an hour, till the bowels have been distinctly acted on by the medicine, and then a pill containing one grain of opium is to be taken.

Even where the pain is considerable, and the case threatens to be severe, this simple plan often succeeds.

*Northern Journal of Medicine, October*, 1844.

